# Emotion-Adaptive Large Language Model–Driven Clinical Decision Support: User Evaluation of the Empathic Clinical Decision Support System Framework for Trust and Explainability

**DOI:** 10.2196/89005

**Published:** 2026-05-22

**Authors:** Tongze Zhang, Sang Won Bae, Tammy Chung, Anind K Dey

**Affiliations:** 1 Department of Systems Engineering, Human-Computer Interaction and Human-Centered AI Systems Lab, AI for Healthcare Lab Charles V. Schaefer, Jr. School of Engineering and Science Stevens Institute of Technology Hoboken, NJ United States; 2 Institute for Health, Healthcare Policy and Aging Research Rutgers Biomedical and Health Sciences Rutgers University Newark, NJ United States; 3 Information School University of Washington Seattle, WA United States

**Keywords:** cannabis intoxication, cannabis-intoxicated behaviors, personalized intervention, large language models (LLMs), algorithmic decisions, transparency, healthcare AI, trustworthy AI, facial emotion recognition, mobile phone, artificial intelligence

## Abstract

**Background:**

The increasing prevalence of cannabis use has motivated researchers to develop computational behavioral models that predict usage patterns and related health impacts in naturalistic environments. However, the opaque nature of many artificial intelligence (AI) systems limits users’ ability to interpret outputs and undermines trust. Existing explainable artificial intelligence techniques often remain overly technical and do not account for the confusion or frustration clinicians may experience when interpreting complex model explanations.

**Objective:**

We propose and evaluate an empathic clinical decision support system (empathic-CDSS) that integrates large language models (LLMs) with real-time emotion recognition and explainability modules. The goal is to provide transparent, adaptive, and emotionally attuned explanations that enhance interpretability, user confidence, and trust in AI-assisted clinical decision-making.

**Methods:**

Our empathic-CDSS integrates explainable artificial intelligence, causal inference, and affective computing within an interactive LLM-driven framework. Users’ affective states were inferred using the circumplex model of affect, which characterizes emotions along 2 dimensions: valence (the degree of pleasure or displeasure) and arousal (the level of physiological activation or energy). Based on the captured emotional signals, the system dynamically adjusts the tone, structure, style, and complexity of its explanations generated by a fine-tuned LLM, enabling personalized and plain-language explanations. A total of 33 participants with diverse medical and technical backgrounds engaged with the system through guided evaluation tasks and postsession assessments to evaluate 6 dimensions of user-centered questions: usability, personalization and relevance of insights, clarity and comprehensibility, system benefits, satisfaction, and trust and reliability.

**Results:**

Our empathic-CDSS effectively generated personalized and transparent explanations that revealed the causal reasoning behind model predictions while enhancing users’ emotional engagement and trust in the system’s decision logic. Continuous, affect-based feedback enabled the system to dynamically adapt explanation delivery to individual user needs. Participants reported significantly improved usability, clarity, satisfaction, and trust compared with a baseline clinical decision support system without emotion adaptation. Improvements were also observed across additional evaluation dimensions, including personalization and perceived system benefits, supporting the feasibility and added value of integrating empathy-aware communication into AI-driven clinical decision support.

**Conclusions:**

This study introduces a transparent, trustworthy, and emotionally adaptive framework for AI-assisted prediction and clinical decision support. By uniting causal reasoning, affective sensing, and LLM-based natural-language explanations, the empathic-CDSS offers a novel direction for developing emotionally intelligent and user-centered AI systems, with potential applications in behavioral monitoring and personalized interventions, including cannabis use and related health domains.

## Introduction

The integration of artificial intelligence (AI) and machine learning (ML) into health care has significantly advanced the development of clinical decision support systems (CDSSs) that can analyze complex health behaviors such as cannabis use. By combining multimodal data, including physiological, behavioral, and contextual information, AI models can provide real-time insights that assist clinicians in monitoring patient risks and treatment response. However, despite these advances, many existing CDSS systems still face challenges in terms of transparency and interpretability, and these issues remain key obstacles hindering their effective clinical application [1].

Given these limitations, explainable artificial intelligence (XAI) has been increasingly adopted to reveal how predictive models generate outputs (eg, feature-importance visualization, counterfactual explanations, and causal modeling). Yet, in practice, XAI outputs are often presented through technical plots, numeric weights, or attribution scores that clinicians without AI training may struggle to interpret. This can increase cognitive load and trigger negative affect, which paradoxically reduces trust in systems intended to improve transparency.

Despite rapid advances in XAI methods, a critical bottleneck persists in explanation delivery: how to convey model reasoning processes in practical clinical interpretation tasks in a manner that nontechnical clinicians can easily understand and use, rather than merely generating technically correct explanations. Cognitive factors such as mental workload and information complexity, as well as emotional factors such as trust, frustration, and confidence in AI recommendations, can significantly influence how clinicians interpret and rely on AI-generated insights.

Driven by the demand for more human-centered design, recent research has begun exploring how emotional and cognitive factors influence interactions between clinicians and AI systems. Several lines of research have attempted to improve human-AI interaction in CDSSs through better communication design and adaptive data presentation. For example, integrating sensor data from wearable and mobile devices has enhanced the ability of CDSSs to detect and predict patient health states in real time [1-4]. In parallel, advances in large language models (LLMs) have made it possible to process unstructured data and generate natural-language explanations [5-10]. Within the human-computer interaction community, LLM-driven conversational agents have been explored to improve medical reasoning, facilitate clinician-AI dialogue, and make AI-generated insights more accessible. However, despite their potential, most existing systems provide static, one-size-fits-all explanations that do not adapt to users’ emotional or cognitive states. This limitation constrains their ability to sustain user engagement and trust, especially in emotionally demanding clinical contexts. Existing systems often treat interpretation as a fixed content generation process, underestimating health care professionals’ tolerance for complexity and the dynamic shifts in emotional engagement and trust during moments of uncertainty, stress, or time pressure.

Emerging evidence from affective computing suggests that detecting and responding to users’ emotions can substantially improve perceptions of system usability, reliability, and trustworthiness [11-14]. Negative emotions such as confusion or stress can reduce users’ ability to comprehend complex outputs, whereas emotionally attuned interactions can promote engagement and confidence in AI systems. These dynamics are particularly relevant in health care, where clinicians must make rapid judgments under pressure and cognitive load. Complementary research in algorithmic empathy has shown that conversational LLMs can simulate empathetic dialogue. For instance, ChatGPT (OpenAI) has been observed to express empathy more often than physicians in patient-facing scenarios [15]. Nevertheless, such “synthetic empathy” can lack contextual awareness or amplify biases learned during training [16,17]. Moreover, most empathy-oriented AI systems rely solely on text-based sentiment analysis, overlooking multimodal affective signals such as valence (emotional pleasantness) and arousal (physiological activation) that form the foundation of real-time emotion modeling [18,19]. These limitations indicate a gap in developing CDSSs that dynamically adapt their communication based on clinicians’ emotional states during AI interaction. To address these challenges, we developed the empathic clinical decision support system (empathic-CDSS), a novel framework that integrates XAI, affective computing, and LLMs to create an emotion-adaptive, explainable dialogue system for clinical decision-making. The system incorporates three core components: (1) a multimodal emotion-recognition module that analyzes facial behavior and linguistic sentiment to infer users’ valence and arousal in real time, (2) an XAI engine that uses SHAP (Shapley Additive Explanations), causal reasoning, and counterfactual analysis to generate transparent model explanations, and (3) an adaptive dialogue module powered by a fine-tuned LLM that dynamically adjusts the tone, complexity, and level of detail in its explanations based on the detected affective state. Through this integration, the empathic-CDSS aims to bridge the gap between technical explainability and emotional alignment, providing explanations that are both transparent and contextually sensitive to the user’s experience. Based on this framework, this study aims to investigate whether such emotion-adaptive interpretability can enhance clinicians’ trust, usability, and comprehension of AI-generated insights.

After developing the empathetic CDSS, we evaluated its impact on user perception and interaction experience. This study evaluates whether the empathic-CDSS enhances trust, usability, and interpretability among users with diverse clinical and technical backgrounds. Specifically, we asked: (1) How can real-time emotion recognition be integrated into an explainable dialogue system to guide adaptive responses during user-AI interaction? (2) How does emotion-adaptive explanation influence users’ trust, satisfaction, and perceived reliability compared with a baseline CDSS? (3) How does the integration of personalized and real-time affective state awareness-based feedback impact the usability, interpretability, and accessibility of XAI for multidisciplinary users (clinicians and technical users)?

This study contributes to both the design and evaluation of emotion-adaptive XAI for clinical decision support. Specifically, it (1) introduces a novel framework (empathic-CDSS) that unites XAI, affective computing, and LLM-driven dialogue for empathic human-AI interaction; (2) demonstrates how valence-arousal emotion modeling can inform dynamic adaptation of AI explanations in real time; and (3) provides empirical evidence that emotion-adaptive explainability enhances trust, usability, and interpretability in clinical AI tools.

The remaining sections are organized as follows. The Methods section describes the empathic-CDSS architecture, participant recruitment, and evaluation procedure. The Results section reports quantitative and qualitative findings on usability, trust, and system satisfaction. The Discussion section interprets key results in the context of prior work and implications for empathic AI design, and the conclusions summarize contributions and directions for future research.

## Methods

### Study Design

We conducted a mixed-methods, within-subject experiment to evaluate the usability, trust, and explainability of two CDSS versions: (1) a baseline CDSS providing static explanations without emotion perception, and (2) an empathy-enhanced version integrating real-time emotion perception and adaptive explanation generation.

We designed and evaluated a novel CDSS named empathic-CDSS. The core functionality of empathic-CDSS integrates (1) analyses of multimodal patient data (eg, sensor data and self-report), with (2) real-time multimodal emotion recognition of the user who is using empathic-CDSS to deliver to the user, more transparent, empathic, and understandable explanations of the model’s results.

For analyses of data provided by the “patient,” the system is configured to analyze passive sensor data collected via smartphones and wearable devices to predict specific behavioral outcomes for the patient (eg, whether a cannabis user is in an “intoxicated” state). The system then uses multiple XAI techniques (including SHAP [20], causal analysis [21], and counterfactual reasoning) to provide explanations for these person-level predictions [22]. For further technical details on the model generation pipeline and the XAI-based interpretability results, please refer to Multimedia Appendix 1, particularly the sections titled Personalized Model Generation for Cannabis Users (“Patients”) and Interpretability Analysis.

For the health care provider, or clinician or researcher who needs to interpret empathic-CDSS model outputs (ie, person-level prediction models), empathic-CDSS innovatively integrates, in real-time, multimodal emotion perception functionality as the user uses the system. Specifically, the system analyzes, in real time, users’ facial expressions (extracting valence and arousal) and emotions from dialogue text entered in the system (eg, sentiment analysis), to capture users’ emotional states. The system uses emotional insights from the user’s facial expressions and dialogue text to dynamically adjust the style and complexity of the system’s explanations of model outputs through a fine-tuned LLM, thereby providing a more empathetic and personalized interactive experience for the user (eg, the user using the system).

### Participants

A total of 33 participants (P1-P33) took part in this study to represent the user who needs to interpret the empathic-CDSS model outputs. Participants were predominantly male (approximately 20/33, 70%), a majority aged 20-29 years, and most were highly educated (20 PhD students, 7 master’s students, and several professionals such as physicians, nurses, and medical product managers). Participants were recruited through online advertisements and community networks. Eligibility criteria included adults aged 20 years and older. No age-based exclusion criteria were applied. Age distribution reflects voluntary enrollment during the recruitment period. Participants represented diverse disciplines, including engineering, computer science, psychology, and health care. Participants’ experience in the medical field ranged from none to more than 20 years, providing a balanced mix of novice and expert perspectives for evaluating the explainable and emotion-aware AI system. This heterogeneous sample of system users was intentionally targeted to capture the interdisciplinary ecosystem in which clinical AI tools are typically developed and deployed, spanning both technically trained developers and nontechnical health care professionals. Including participants with varying levels of technical and clinical expertise allowed examination of differences across users in interpretability, emotional receptivity, and usability perceptions, thereby ensuring that the system’s design and evaluation reflect real-world diversity among potential end users in clinical and decision-support contexts. Detailed demographic information about the participants is provided in Table 1.

**Table 1:** Participant demographics and professional background (N=33).

Characteristic and category			Values, n (%)
**Gender**			
	Man	20 (60.6)	
	Woman	10 (30.3)	
	Prefer not to say	3 (9.1)	
**Age (years)**			
	20-29	20 (60.6)	
	30-39	6 (18.2)	
	50-60	7 (21.2)	
**Education level**			
	Bachelor’s	3 (9.1)	
	Master’s	9 (27.3)	
	PhD student	17 (51.5)	
	PhD	4 (12.1)	
**Occupation or discipline**			
	PhD or master’s student (engineering, computer science, information systems, or psychology)	20 (60.6)	
	Medical product manager	4 (12.1)	
	Clinician or medical expert (physician, nurse, or counselor)	4 (12.1)	
	Researcher or research assistant	3 (9.1)	
	Other or unspecified	2 (6.1)	
**Experience in medical domain (years)**			
	None (0)	11 (33.3)	
	1-5	5 (15.2)	
	6-10	6 (18.2)	
	11-20	8 (24.2)	
	>20	3 (9.1)	

### Ethical Considerations

This study received approval from the Stevens Institute of Technology Institutional Review Board (2024-090 (N)) before recruitment and data collection began. Written informed consent was obtained from all participants. Participants who completed this study received a US $20 Amazon gift card as compensation.

In accordance with IRB requirements, participation was entirely voluntary. Subjects were informed of their right to withdraw from the laboratory session at any point without any negative consequences, though full completion was required for the compensation.

To protect participant privacy, all collected data (including survey responses and system logs) were anonymized immediately after collection. Personal identifiers were replaced with unique alphanumeric codes (e.g., P01, P02).

All research data were stored on secure, password-protected servers within the laboratory. Access was restricted to the primary research team to ensure that no data would be disclosed to third parties or leaked outside the institution.

### Study Setting and Procedure

This study used a within-subjects design, in which each participant interacted with both versions of the system to enable direct comparison. To mitigate potential learning or order effects, the presentation order of the 2 systems was counterbalanced: approximately half of the participants were randomly assigned to use the basic system first, while the other half used the advanced system first.

This study included 2 experimental conditions. The first was the basic-CDSS, which included the core study features, namely a personalized prediction model for cannabis intoxication and multiple XAI visualization charts (such as SHAP plots and causal diagrams). The dialogue interface was driven by a standard, not fine-tuned GPT-4 (OpenAI) model, and crucially, it did not possess any emotional perception capabilities, so its responses were purely information-oriented and did not adjust based on the user’s emotional state. The second was the empathic-CDSS, which represents the complete empathic-CDSS proposed in this paper. Empathic-CDSS builds upon all the features of the basic-CDSS and integrates two core enhancement modules: (1) a multimodal emotion perception module for real-time analysis of users’ facial expressions and textual emotions; and (2) an empathy dialogue model, which has been fine-tuned on the motivational interviewing (MI) dataset [23] to generate emotion-adaptive responses.

**Figure 1:**
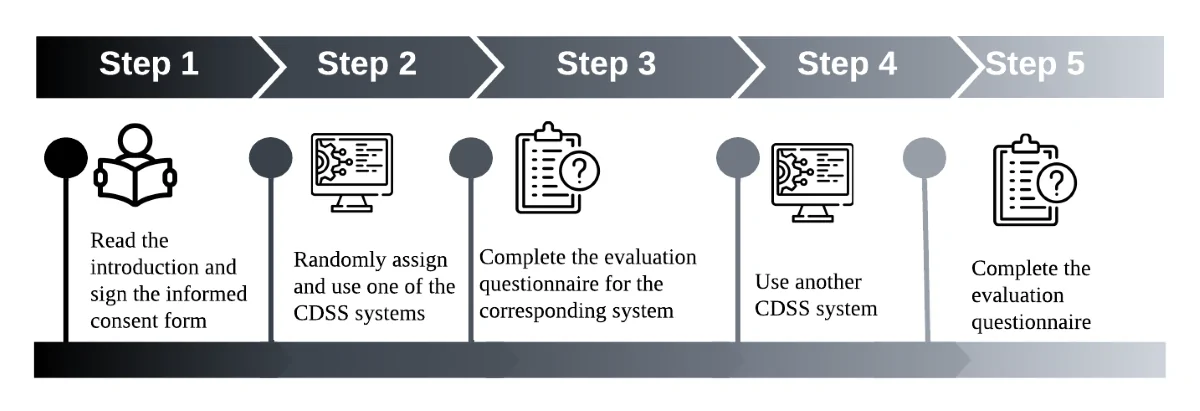
User research tasks and questionnaire evaluation process. CDSS: clinical decision support system.

Before any interaction with the system, participants were asked to review and complete an informed consent form that outlined this study’s purpose, procedures, and their rights as participants. Participation was contingent upon signing this form. The participant then interacted with 1 of the 2 systems (randomly assigned). After completing their interaction with the system, the user filled out a comprehensive posttest questionnaire to evaluate their experience. The user then repeated the same process with the second system and filled out another evaluation questionnaire. At the end of the experiment, we asked participants to complete open-ended questions to collect qualitative feedback on the user experience for both systems. The user study procedure is illustrated in Figure 1.

### System Description: Empathic-CDSS Architecture and Processes

#### Basic-CDSS

The basic CDSS in this study serves as a control system, which aims to provide a standard, data-driven clinical decision support platform. The system primarily relies on traditional explainable ML analysis workflows, including structured data processing, clinical feature extraction, and interpretable presentation of model prediction results. Basic-CDSS uses generic XAI methods to explain model outputs, enabling users to understand the key factors behind predictions and use this information to inform clinical judgments. Compared to the empathic-CDSS developed in this study, basic-CDSS does not incorporate emotion recognition modules (such as text sentiment analysis or facial expression recognition) and does not use model fine-tuning with emotional strategies. Consequently, system interaction uses a standardized, nonemotion-adaptive interface presentation. Nevertheless, the system retains core analytical capabilities, including clinical data processing, model execution, and visual presentation of interpretive results.

#### Empathic-CDSS

The empathic-CDSS developed in this study aims to provide users with an intelligent interactive platform that integrates emotional understanding with XAI analysis. To do this, empathic-CDSS combines XAI methods with emotion-aware interaction design. The system combines multimodal emotion recognition involving text sentiment analysis and facial expression recognition with personalized, explainable ML models. Through this architecture, the system not only generates accurate and transparent analytical results but also presents them through empathetic, emotionally adaptive conversational interactions. To generate and support strategies for empathic responding during the conversational interaction, an MI dataset [23] was cleaned and used for web-based fine-tuning. This approach enhances users’ trust in the system and improves their overall interaction experience.

In order to enable real-time emotion recognition for users interacting with the system and to allow ML models to process facial expressions quantitatively, the raw video data must first be converted into structured numerical features. These numerical representations enable the system to capture facial movements and emotional signals in a format suitable for computational analysis and subsequent modeling of emotion-aware interactions.

For real-time emotion recognition of the person while they are using the system, we first decoded the video frame-by-frame to convert the video into a sequence of still images. Each decoded frame was processed individually using our FacePsy [24] code to extract facial action units, head pose parameters (yaw, pitch, and roll), confidence scores for 7 emotion categories (angry, disgust, fear, happy, sad, surprise, and neutral), and 68 facial landmarks [20]. Specifically, action units are a set of features defined by the Facial Action Coding System that are used to quantitatively describe the activity of facial muscles. In addition, we extracted the confidence distribution of 7 basic emotions from the image: angry, disgust, fear, happy, sad, surprise, and neutral. These emotion confidences are inferred directly from facial features using a deep learning model, reflecting the likelihood of multiple emotions in each frame. To describe the geometry of the face, we used standard facial landmarking techniques to extract 68 facial landmarks (landmark0 to landmark67). These landmarks cover the precise locations of facial boundaries, eyebrows, eyes, nose, mouth, etc, providing a basis for capturing expression and emotion changes. The numerical combination of these features forms a high-dimensional feature vector for further analysis and modeling. Examples of facial behavior features are provided in Multimedia Appendix 2.

**Figure 2:**
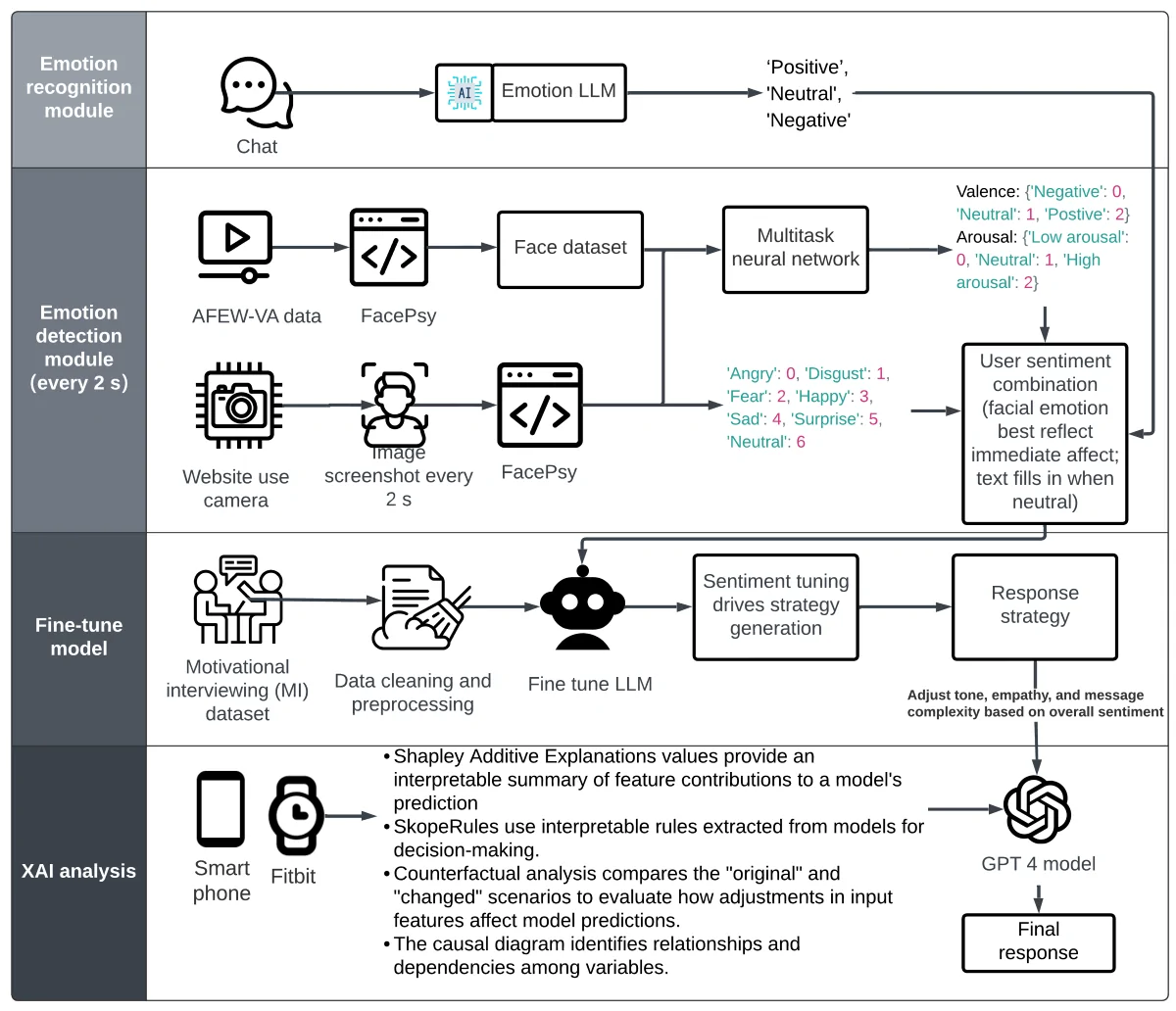
Empathic-CDSS architecture and processes. AFEW-VA: Acted Facial Expressions in the Wild – Valence-Arousal (a publicly available dataset for affective computing); CDSS: clinical decision support system; LLM: large language model; MI: motivational interviewing; SHAP: Shapley Additive Explanations; XAI: explainable artificial intelligence.

Figure 2 shows the overall architecture and data processing flow of this system. Using data from smartphone and wearable device (eg, Fitbit [Google LLC]) collected in a separate study from individuals who use cannabis [25], the system combined several explanatory AI models (eg, SHAP, SkopeRules, and counterfactual analysis) and causal analysis to generate the corresponding explanatory analysis [20-22,26]. The process involves user or clinician (hereafter: “clinician”) input to the system (eg, asking the system a question), AI model processing, interpretable analysis generation, and finally, feedback provided to the user.

In order to keep track of the user’s interaction history and analysis results, the system is designed with a data persistence mechanism. Each user input, system-generated response, and corresponding timestamp are recorded and stored. This not only supports the system’s session retention function but also provides valuable historical data for subsequent data analysis and improvement.

#### Measures

Participants completed a structured evaluation comprising 6 dimensions: system usability [6], personalization and relevance [27], clarity and comprehensibility [5], system benefits [3], overall satisfaction [9,16], and trust and reliability [17,28]. Table 2 shows the constructs for each dimension. Each item was rated on a 10-point Likert scale (1=lowest, 10=highest). Example items include “How easy was it to interact with the system using natural language?” and “How clear and understandable were the explanations provided by the system?” The complete questionnaire is available in Table 2 and Multimedia Appendix 3.

**Table 2:** Survey constructs and example items.

Section	Construct measured	Example item (scale range: Likert 1-10)	References
System usability	Ease of interaction, intuitiveness, and satisfaction	“How easy was it to interact with the system using natural language?”	[6]
Personalization and relevance	Perceived individualization of system insights	“To what extent did you feel the insights were personalized to patient data?”	[27]
Clarity and comprehensibility	Understandability of explanations and visuals	“How clear and understandable were the explanations provided by the system?”	[5]
System benefits	Perceived usefulness and outcome impact	“How beneficial do you believe the insights were in improving understanding of patient health behaviors?”	[3]
User satisfaction	Satisfaction	“How satisfied are you with the overall quality of the insights and recommendations provided by the system?”	[9,16]
Trust and reliability	Trust and perceived reliability	“Please rate the perceived trustworthiness of this system in meeting your needs.”	[17,28]

### Data Collection

#### Cannabis Use Data from “Patients”

We used the AWARE framework app [29] to track cannabis users’ smartphone sensor data, such as GPS and accelerometer, alongside Fitbit Charge 2 data for heart rate and step count. The data were analyzed to identify patterns of cannabis use, with physiological and behavioral data segmented into 5-minute intervals. Key statistics, such as heart rate and step count, were extracted to evaluate cannabis intoxication. Events were classified into “intoxicated” and “not intoxicated” to test the system’s ability to predict and interpret behavioral outcomes from sensor inputs [25]. These ground truth labels were obtained through daily ecological momentary assessment reports, where participants self-reported their current intoxication status upon receiving a prompt on their smartphone. This dataset comprises passively collected smartphone and wearable device data linked to self-reported cannabis intoxication episodes. After preprocessing, the data were segmented into annotated samples representing intoxicated and nonintoxicated states. As the data was gathered in natural settings, the class distributions reflect real-world reporting patterns rather than artificially balanced designs. To ensure model training consistency and robustness, standard preprocessing steps were implemented, including normalization, missing value handling, and participant-level modeling.

#### User Chat Data With the CDSS

For the chat data, user interactions, and generated responses with the CDSS were logged in Google Sheets (Google LLC). The data were structured with fields for email, timestamp, role (clinician or CDSS assistant), and content [30]. This structure allows for persistent storage of conversation history, which can be retrieved and used to maintain context in the ongoing session.

### Data Analysis

#### Quantitative

The quantitative data for this study were derived from clinical practitioners’ ratings of the systems on a 1-10 Likert scale following their experience with both the baseline-CDSS and empathic-CDSS. The rating scale encompassed multiple dimensions, including system usability, personalization and relevance, clarity of explanations, system benefits, user satisfaction, and trust and reliability. As the same group of users evaluated both systems, paired *t* tests were conducted for each questionnaire item to assess differences between the empathic-CDSS and baseline-CDSS scores. For each item, the program automatically aligned valid paired samples across both systems, calculating the mean score, SD, *t* statistic, and *P* value for both empathic-CDSS and baseline-CDSS.

#### Qualitative

Open-ended questions in the questionnaire were collected under the empathic-CDSS condition to supplement users’ subjective experiences and perceptions of the system. These responses to open-ended questions primarily serve a descriptive purpose and summarization, aiding discussions of quantitative findings by illustrating how users interpret the system’s explanatory approaches, personalized presentations, and emotion-adaptive behaviors.

### ML or System Evaluation

At the natural language processing and dialogue generation level, both systems support users posing questions to the CDSS using natural language. The system first performs semantic parsing of user input, mapping natural language into retrieval requests for backend models and data stores. It extracts intent categories and slot information related to patient timeframes and metric types. Empathic-CDSS further incorporates sentiment analysis and emotion-adaptive modules. It uses a sentiment classifier based on pretrained language models to identify emotions in input text, outputting sentiment polarity and coarse-grained emotion categories (eg, neutral, confused, and concerned) as additional conditions for dialogue planning. Subsequently, the dialogue generation module integrates backend ML predictions, XAI explanations (eg, SHAP feature contribution rankings, key curves, and temporal trend charts), and sentiment analysis signals to generate responses that align with model outputs while adapting to the user’s current emotional state: when detecting higher uncertainty or stress, the system uses more step-by-step, detailed explanatory structures and a more supportive tone; while using more concise, summary-style answers during neutral or positive emotional states. Baseline-CDSS uses the same cannabis intoxication prediction and XAI pipeline but presents results in a uniform, nonemotion-adaptive format, excluding sentiment analysis or linguistic style adaptation. XAI model reliability is evaluated by separating smoking session data, with a portion used for model testing and the remaining samples for model training. Predictions are validated within retained sessions to ensure stable personalized performance, thereby enabling interpretable generation for CDSS.

### Model of Emotion Valence and Arousal

We constructed an efficient model training framework for multiclassification problems with dual labels. All samples in the dataset were labeled, and the objective was to improve the training efficiency and generalization ability of the model while ensuring classification performance. Due to the large size of the training set, using all samples directly may cause the model to be disturbed by redundant information during the learning process. Therefore, we systematically designed the model training process through cross-validation (5-fold cross-validation) and feature preprocessing to improve model performance and robustness.

In the data preprocessing stage, we cleaned and converted missing values, nonnumeric fields, and coordinate-type features in the data. Coordinate information was parsed from string format into numeric vectors and expanded into separate feature columns. Subsequently, we standardized all input features to eliminate the influence of different units of measurement and ensure that the model had stable gradients and convergence speed during training.

This study used a multitask neural network (multitask neural network structure) [31] to simultaneously perform 3-class classification prediction for 2 emotion dimensions, valence (negative, neutral, and positive) and arousal (low arousal, neutral, and high arousal) [32]. This structure introduced shared hidden layers, enabling the model to share representation learning between 2 related tasks, thereby improving training efficiency and enhancing generalization ability.

The shared feature extraction part consists of 2 fully connected networks, combined with batch normalization, ReLU (rectified linear unit; an activation function commonly used in deep learning models, including facial recognition systems) activation functions, and dropout, which can effectively enhance the model’s expressive ability while reducing the risk of overfitting [33]. The 2 output branches use independent fully connected layers to predict the arousal and valence labels, respectively.

During training, we used the cross-entropy loss function [34] to supervise each output branch. The final optimization objective was the weighted sum of the 2 losses, expressed as follows: *L*=*λ*_1_*L*_(_*_Arousal_*_)_+*λ*_2_*L*_(_*_Valence_*_)_, *L*_(_*_Arousal_*_)_ and *L*_(_*_Valence_*_)_ represent the cross-entropy losses for arousal and valence, respectively. This structure demonstrates robust performance and classification accuracy when handling 2 emotional label tasks simultaneously.

We used the Adam optimizer to minimize this loss function during training [35]. The model updates parameters through backpropagation and iteratively optimizes the performance of the 2 tasks. This structure demonstrates good efficiency and performance in multilabel multiclassification problems, capturing both shared information between tasks and task-specific discriminative features.

### Fine-Tuning Model and Dual Language Model Architecture

To achieve more empathic and context-aware responses, the system uses a dual language model architecture that leverages both a standard GPT-4 model [36] and a fine-tuned GPT-4 model [37]. All LLM functionalities in this study, including standard GPT-4 calls and model fine-tuning, were implemented via the official application programming interfaces provided by OpenAI. This design ensures that the system combines XAI analysis with emotional context for a comprehensive and user-centered interaction. The first language model is the standard GPT-4, which is used to process the output generated by the XAI component. The model interprets the results of XAI analyses, such as feature importance, counterfactual reasoning, or causal inference graphs, and extracts key insights. The second language model is a fine-tuned GPT-4 that has been specifically trained using an MI dataset [23]. This MI dataset provides the basis for equipping the model with the ability to recognize and respond to users’ emotional states. The fine-tuning process enables the model to generate responses that adhere to the principles of communicating empathy and support, based on the results of the sentiment analysis. This is crucial for building engagement into the interaction.

**Figure 3:**
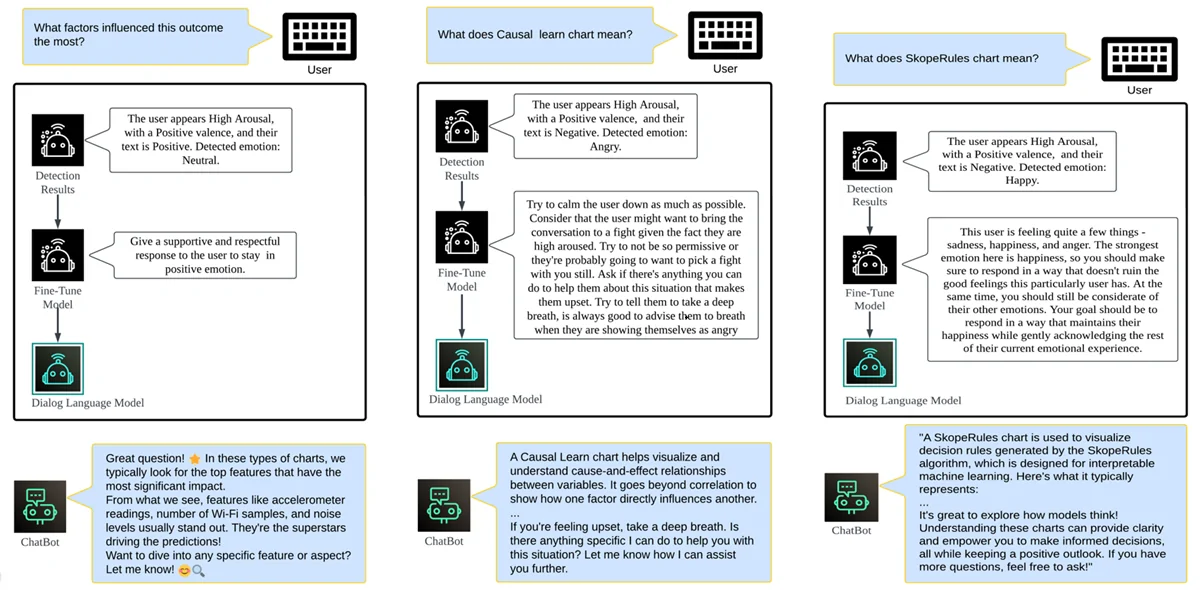
Examples of emotion detection language model.

The fine-tuning process included preprocessing the dataset to ensure data quality and compatibility with the GPT architecture. Noise was eliminated, annotations of emotional states were aligned, and tokenization was optimized to preserve empathetic communication. These steps enabled the model to learn conversational patterns that emphasize understanding, validation, and support. Through this process, the model generates responses that match the user’s psychological needs based on the emotional valence and arousal, as well as the sentiment of the user’s text. The fine-tuning process focuses on enabling the model to recognize subtle emotional cues in user input and generate responses that adhere to the principles of empathy and support. By incorporating real-world patterns of conversation from the MI dataset, the system learns to address the user’s concerns in a compassionate manner, which promotes engagement. This fine-tuning approach enhances the system’s ability to dynamically adapt to different emotional contexts, ensuring that the user not only receives accurate explanations, but also emotionally resonant responses that make interactions more human-centered. Fine-tuned by integrating a corpus of MI data, the system now strikes a good balance between empathetic communication and clear explanations. This approach not only improves the user’s experience but also reinforces the trustworthiness of the system as an XAI interaction. The system integrates multiple high-level components, including emotion recognition and natural language processing models, to achieve its core functionality. One model detects the user’s emotional state through a combination of text sentiment analysis and facial expression recognition. Then, another model generates personalized and context-aware responses based on the detected emotion, as shown in Figure 3.

### Dialog System

**Figure 4:**
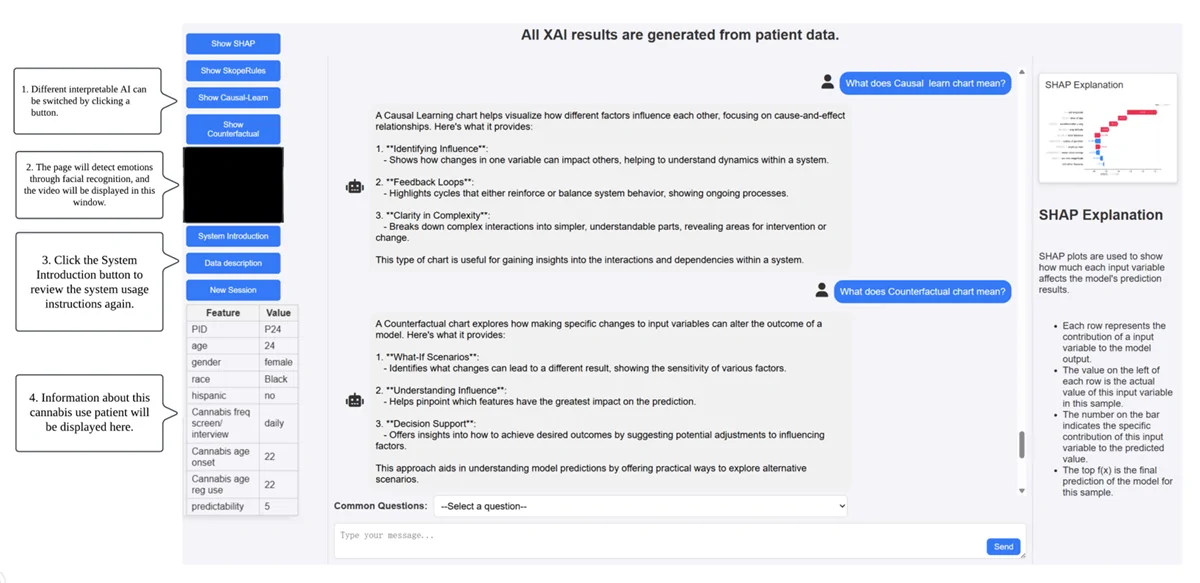
Empathic-CDSS demonstration. AI: artificial intelligence; CDSS: clinical decision support system; PID: participant ID; SHAP: Shapley Additive Explanations; XAI: explainable artificial intelligence.

The system provides an intuitive way to present complex data analyses directly to the user. Data tables are presented visually in the interface, eliminating unnecessary navigation steps. To facilitate understanding, the contents of these tables are displayed immediately without additional manipulation. The system automatically converts the analyzed data into visualizations such as SHAP plots, cause-effect plots, and rule interpretation plots. These graphical outputs greatly enhance the clarity of the information, while the images are encoded in base64 and embedded in the web page to enrich the visual experience [38]. Each table is accompanied by a detailed explanation, including the names of the features and their respective importance or influence. In addition, the system can seamlessly convert the XAI results in the data table into images and embed them in the user interface. This approach not only improves the readability of the data, but also helps the user understand the analysis results in a more intuitive way. The system interface is shown in Figure 4.

The system allows users to interact via a natural language interface, and each message is subject to a multistep sentiment analysis. The system uses 3 different mechanisms to assess the emotional state of the user. First, the sentiment of the input text is analyzed and classified as positive, negative, or neutral. At the same time, when the camera is active under the empathic-CDSS condition, facial expression recognition is used to assess the visible emotions of the user. In addition, indicators of emotional valence and arousal are calculated to capture the intensity and type of emotion, providing a comprehensive understanding of the user’s emotional context.

We use a dual model architecture to process the results of 3 sentiment analysis methods (text sentiment, facial expression recognition, and value arousal indicators). The fine-tuned model only receives the sentimental results and generates preliminary analysis and corresponding response guidance based on these results. This response guidance is then passed to the GPT model, which takes the user’s question and analysis results as input to generate the final response. This step-by-step approach ensures that the generated response is not only consistent with the sentiment context, but also effectively explains the XAI output in a user-friendly and sentiment-intelligent manner.

To improve the quality of the interaction, the system keeps a detailed dialog history and ensures that the conversation is consistent. The system can adapt its responses based on previous exchanges, making the interaction more personalized and meaningful. Finally, the system presents feedback through a combination of text, visuals, and emotion summaries. These outputs not only provide explanations for the model’s decisions but also include explanatory images or diagrams to help the user understand the system’s behavior intuitively. By integrating text sentiment analysis, facial expression recognition, and value arousal metrics, the system can respond in a user-centered manner.

## Results

### Performance of Personalized Models for Predicting Cannabis Intoxication

We first assessed the performance of the personalized predictive model designed to estimate cannabis intoxication. To approximate real-world prediction conditions, we used a personalized evaluation strategy in which self-reported cannabis intoxication from selected individuals served as the prediction target. A total of 3 representative cannabis users were selected, and an individualized model was trained and evaluated for each user. For each model, 1 day of self-reported “cannabis use” was used as the test set, while all remaining historical data served as the training set.

Across all 3 users, the personalized models demonstrated strong predictive capability, achieving *F*_1_-scores exceeding 70% in independent tests. These results indicate that the models effectively captured individual behavioral patterns and generated reliable predictions of future intoxication states. The prediction outputs and corresponding XAI explanations from these tests were subsequently incorporated into the CDSS interface presented to users during the comparative evaluation of the basic and empathic systems.

### Affective Sensing Model Performance

We next evaluated the performance of the emotion-perception component of the system. Using the AFEW-VA (Acted Facial Expressions in the Wild – Valence-Arousal [a publicly available dataset for affective computing]) dataset [39], we conducted 5-fold cross-validation to assess arousal and valence prediction accuracy. As summarized in Table 3, the model achieved strong overall performance across folds. For arousal prediction, the model achieved an average *F*_1_-score of 0.75 (SD 0.0086) and an accuracy of 88.16% (SD 0.0020). For valence prediction, the average *F*_1_-score was 0.70 (SD 0.1173), with an accuracy of 74.39% (SD 0.0075).

To deploy the affective sensing module within the empathic-CDSS, we selected the best-performing model fold based on overall cross-validation metrics. The detailed performance of this selected fold is presented in Table 4.

As shown in Table 4, the selected model achieved an *F*_1_-score of 0.75 with an accuracy of 88.03% for arousal prediction, and an *F*_1_-score of 0.71 with an accuracy of 74.78% for valence prediction. These metrics confirm the effectiveness and reliability of the affective sensing module integrated into our empathic-CDSS.

**Table 3:** Average performance metrics of the affective sensing model.

Metrics	Arousal	Valence	Average (SD)
*F*_1_-score	0.75	0.70	0.73 (0.035)
Precision	0.82	0.74	0.78 (0.057)
Recall	0.70	0.68	0.69 (0.014)
AUC^a^	0.93	0.87	0.90 (0.042)
Accuracy (%)	88.16	74.39	81.28 (9.75)

^a^AUC: area under the curve.

**Table 4:** Performance metrics of the best fold affective sensing model.

Metrics	Arousal	Valence	Average (SD)
*F*_1_-score	0.75	0.71	0.73 (0.028)
Precision	0.80	0.74	0.77 (0.042)
Recall	0.71	0.70	0.71 (0.007)
AUC^a^	0.93	0.87	0.90 (0.042)
Accuracy (%)	88.03	74.78	81.41 (9.37)

^a^AUC: area under the curve.

### Overall System Performance

We systematically evaluated our emotion-aware approach, which integrates continuous valence-arousal modeling with LLM-based dialog generation. The results indicate that the use of continuous affective representations substantially improves the system’s ability to detect, interpret, and respond to subtle user sentiments during real-time interactions. Compared with traditional classification-based models, our approach enabled more contextually appropriate, emotionally consistent, and empathetic responses.

User feedback also confirmed smoother conversational flow, fewer emotionally inconsistent replies, and improved alignment with user intent. Unlike conventional affective models that depend on fixed emotion categories, our system adapts dynamically to changes in user emotional state, leading to more natural and personalized interactions.

### Quantitative Comparison Between Systems

Multimedia Appendix 3 outlines the evaluation areas included in this study. To quantify differences between the empathic-CDSS and the basic CDSS, we conducted paired-sample *t* tests for all questionnaire items, reflecting within-participant comparisons between the 2 system conditions (Table 5).

**Table 5:** Comparative results across usability and perception metrics between the empathic- and basic-CDSS conditions. All *P* values follow JMIR formatting guidelines; *P*<.001 denotes highly significant differences at the α=.05 level.

Dimension and question		Empathic mean (SD)	Basic mean (SD)	**t* (32)*	*P* value
**System usability**					
	Q1	8.00 (1.64)	6.12 (2.25)	4.178	<.001
	Q2	7.73 (1.38)	5.88 (2.07)	4.245	<.001
	Q3	6.67 (2.35)	5.21 (2.32)	2.784	.009
	Q4	7.61 (1.71)	6.09 (2.24)	3.318	.002
**Personalization and relevance**					
	Q1	7.76 (1.75)	6.39 (1.89)	3.090	.004
	Q2	7.58 (1.46)	5.76 (1.79)	4.942	<.001
	Q3	7.64 (1.41)	5.73 (1.97)	4.955	<.001
**Clarity and comprehensibility**					
	Q1	8.09 (1.53)	6.39 (2.00)	4.430	<.001
	Q2	8.03 (1.59)	6.30 (2.40)	3.713	<.001
	Q3	8.24 (1.20)	6.55 (2.20)	4.033	<.001
	Q4	7.36 (1.75)	5.58 (1.85)	3.835	<.001
	Q5	7.15 (1.72)	6.12 (1.87)	2.489	.02
**System benefits**					
	Q1	8.03 (1.38)	6.61 (1.78)	4.155	<.001
	Q2	7.64 (1.62)	6.39 (2.22)	2.543	.02
	Q3	7.73 (1.40)	6.45 (2.18)	2.950	.006
	Q4	7.27 (2.15)	6.15 (2.29)	2.211	.03
**User satisfaction**					
	Q1	7.76 (1.20)	5.85 (2.00)	4.357	<.001
	Q2	7.64 (1.43)	6.06 (2.00)	3.859	<.001
**Trust and reliability**					
	Q1	7.85 (1.20)	5.91 (1.59)	5.329	<.001
	Q2	7.79 (1.17)	6.12 (1.62)	5.133	<.001

### System Usability

**Figure 5:**
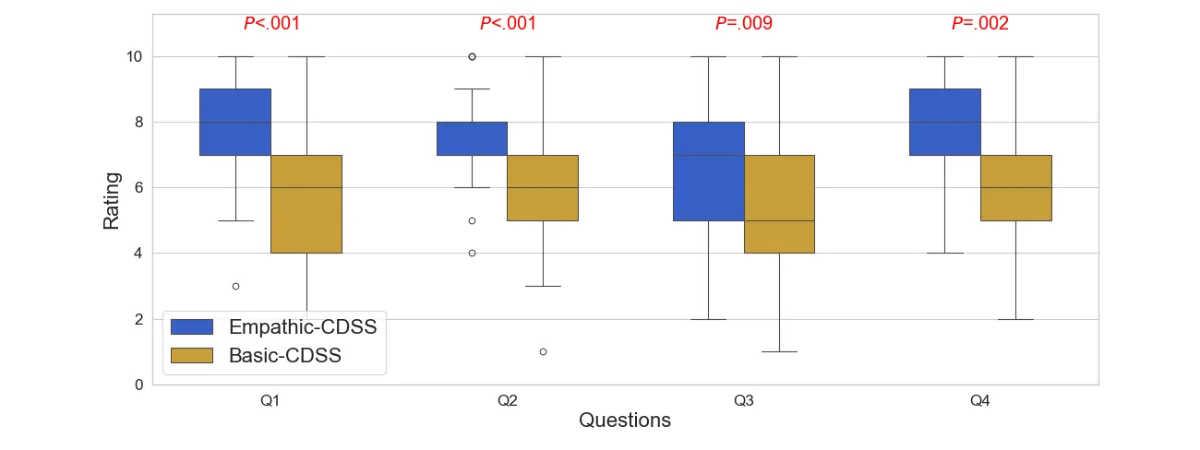
Comparative results across system usability. CDSS: clinical decision support system.

As shown in Figure 5, the empathic-CDSS demonstrated significantly higher usability across all items. For example, users rated the ability to “interact with the system using natural language” higher for the empathic-CDSS (mean 8.00, SD 1.64) compared with the baseline system (mean 6.12, SD 2.25; *P*<.001). Significant improvements were also observed for interface intuitiveness (Q2), response time satisfaction (Q3), and accurate data interpretation (Q4; all *P*<.05). These findings suggest that incorporating emotional-perception feedback results in smoother, more natural clinician-AI interactions.

### Personalization and Relevance of Insights

**Figure 6:**
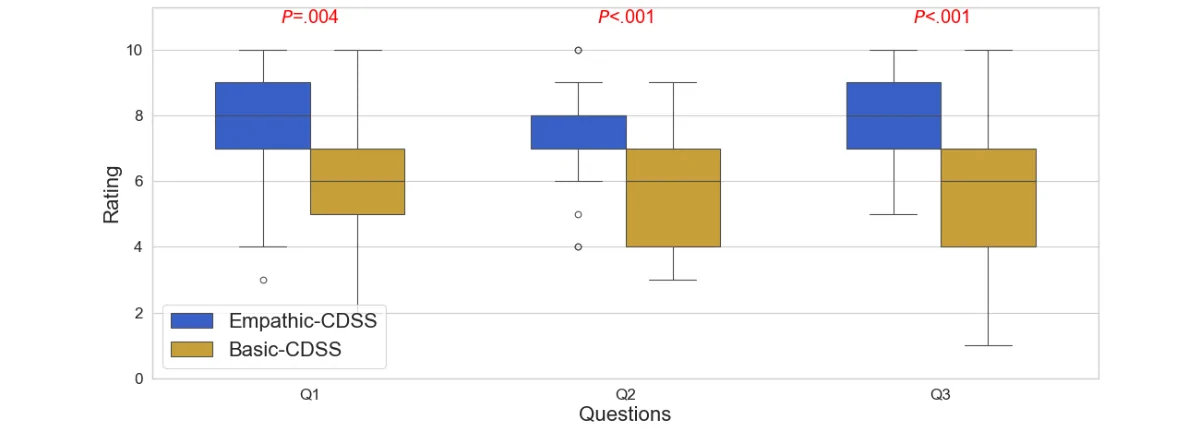
Comparative results across personalization and relevance. CDSS: clinical decision support system.

The empathic-CDSS also outperformed the baseline system in personalization and relevance (Figure 6). For example, users rated the system’s ability to consider the patient’s current condition significantly higher (mean 7.64, SD 1.41 vs 5.73, SD 1.97; *P*<.001). The empathic-CDSS also showed superior performance in handling missing information and referencing appropriate individual-level data (eg, Q5: 7.15 vs 6.12; *P*=.02). These results highlight improved adaptability to patient-specific information and more accurate context-aware responses.

### Clarity and Comprehensibility of Explanations

**Figure 7:**
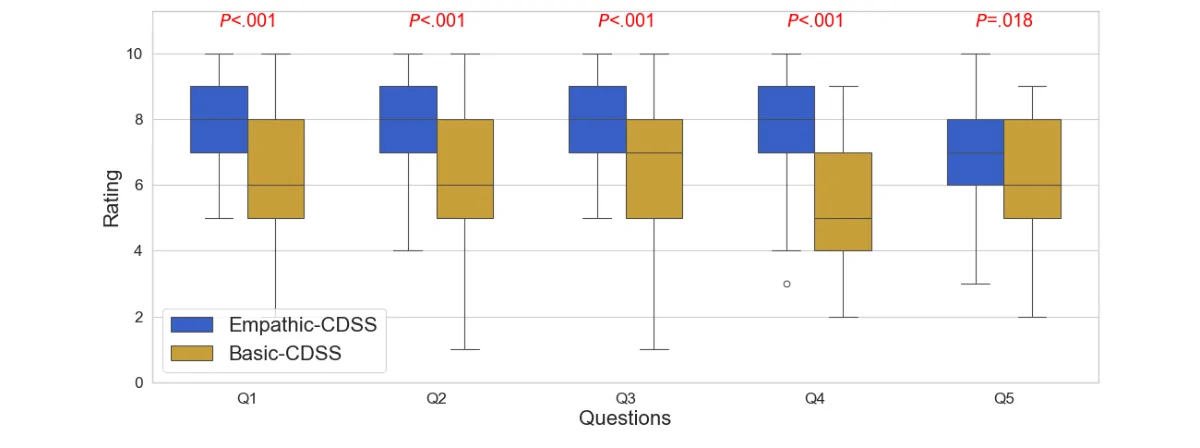
Comparative results across clarity and comprehensibility. CDSS: clinical decision support system.

The empathic-CDSS achieved significantly higher scores (overall mean 7.77, SD 0.49 vs 6.19, SD 0.41) in clarity and comprehensibility compared to the baseline (Figure 7). Participants rated the clarity of explanations provided by the system significantly higher (mean 8.09, SD 1.53, vs 6.39, SD 2.00; *P*<.001). Specifically, the transition from raw technical SHAP data to LLM-generated plain-language narratives helped clinicians better grasp the causal reasoning behind the model’s predictions.

### System Benefits

**Figure 8:**
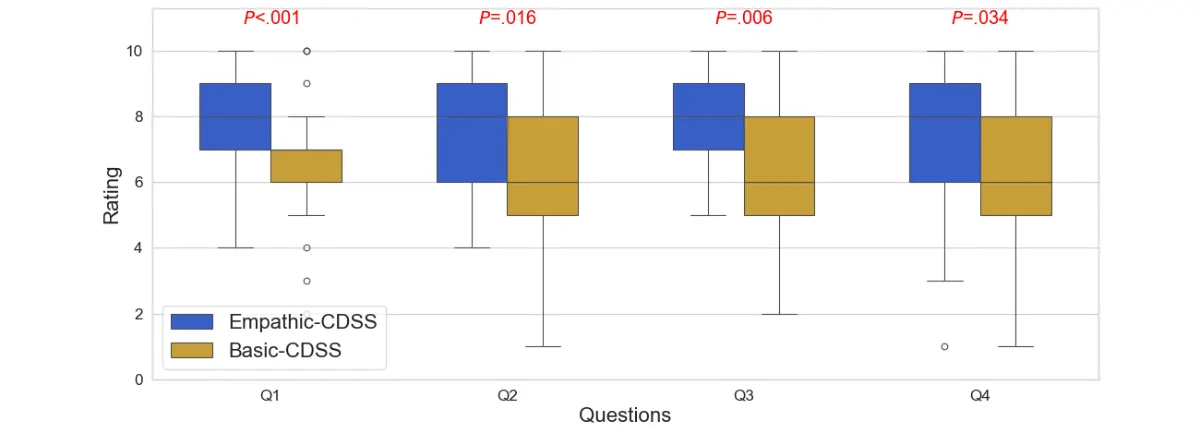
Comparative results across system benefits. CDSS: clinical decision support system.

As shown in Figure 8, the optimized model showed significant advantages across all 4 benefit-related metrics. Participants rated the system as more helpful for understanding patient health behaviors (Q1: mean 8.03, SD 1.38, vs 6.61, SD 1.78; *P*<.001). They were also more likely to recommend the system to others (Q3; *P*=.006). The optimized system further reduced misinformation (Q4), reflecting improvements in output accuracy and clarity.

### User Satisfaction, Trust, and Reliability

**Figure 9:**
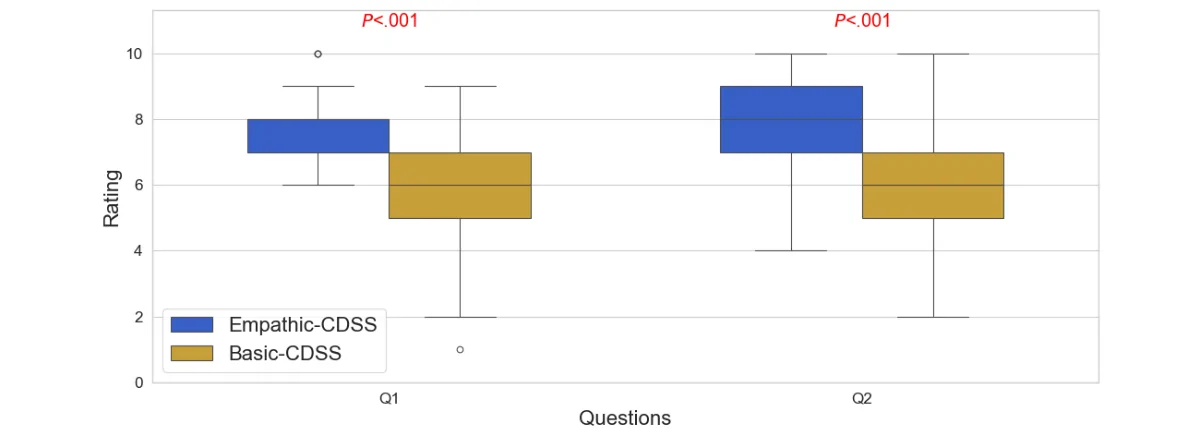
Comparative results across user satisfaction. CDSS: clinical decision support system.

**Figure 10:**
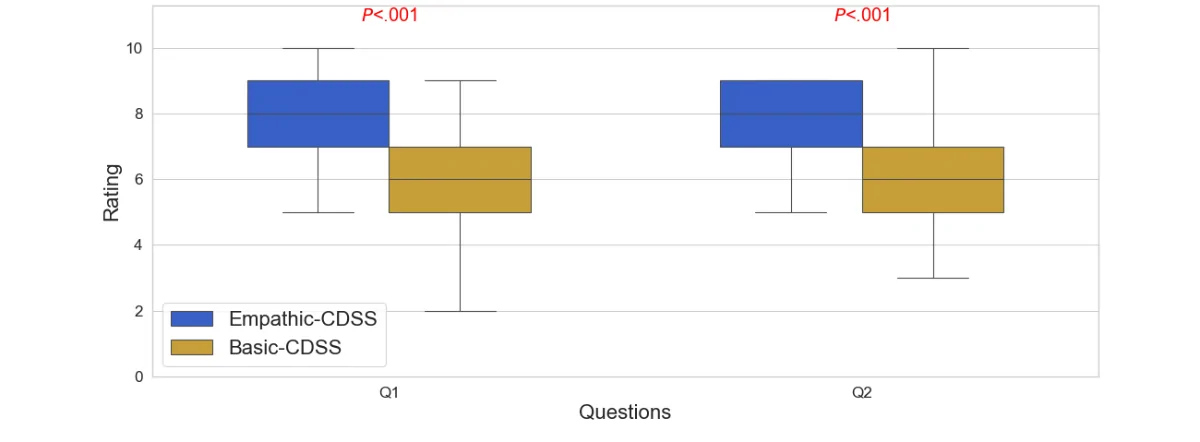
Comparative results across trust and reliability. CDSS: clinical decision support system.

The empathic-CDSS consistently achieved higher satisfaction, trust, and reliability scores (Figures 9 and 10). All satisfaction-related differences were statistically significant (all *P*<.001). For example, users reported a higher likelihood of continued system use (mean 7.76, SD 1.20, vs 5.58, SD 2.00). Trust-related items also favored the empathic-CDSS, with scores above 7.7 on trustworthiness and overall reliability, compared with approximately 6.1 for the baseline model.

### Summary of Findings

Overall, results demonstrate that the empathic-CDSS substantially outperforms the baseline system in usability, interpretability, personalization, trustworthiness, and perceived usefulness. The combination of multimodal sentiment recognition, MI-informed fine-tuning, and XAI-driven decision support significantly enhanced user experience and increased acceptance of the system. These findings emphasize the importance of human-centered AI design and highlight the value of adaptive, affective, and context-aware interactions in improving clinical decision-support performance.

### System Impact on User Emotion and Stress

To assess the systems’ impact on users’ emotional states, we compared self-reported emotion and stress levels before and after interaction with the basic-CDSS and the empathic-CDSS. Most participants reported neutral emotions in both conditions. A small number of mood changes were observed: after using the empathic-CDSS, 1 participant (P14) reported a shift from neutral to happiness, whereas after using the basic-CDSS, 1 participant (P21) reported a shift from happiness to neutral. This same participant showed no mood change when using the empathic-CDSS.

Stress levels also showed modest differences across conditions. Under the basic-CDSS, stress increased for several participants (for example, P21 increased from 2 to 4, and P12 increased from 4 to 6). In contrast, stress levels remained stable or decreased for most participants when using the empathic-CDSS (for example, P22 decreased from 6 to 5, and P31 decreased from 4 to 3). Overall, the basic-CDSS was associated with stress increases in 4 participants and decreases in 7 participants (mean change –0.76, SD 1.51). The empathic-CDSS produced only 1 stress increase, with a similar mean reduction (–0.84, SD 1.73), and stress remained unchanged for most participants.

### Summary of Qualitative Feedback

Users provided generally positive feedback regarding the system’s interactivity, natural language capabilities, and clarity of explanations. Many participants highlighted the system’s ability to support conversational flexibility and deliver rapid, data-driven insights. For instance, P10 praised the system’s natural dialog style, noting: “Any question can be asked, and the system appears to understand.” Similarly, P11 commented that the system “can provide clear and data-driven insights based on the user’s question,” emphasizing trust in its analytical outputs.

Despite this positive reception, users also identified limitations related to explanation depth and interpretability. P11 remarked that “the explanations are not very detailed,” and P13 noted that “the decision path is a bit confusing,” suggesting that the clarity of complex decision pathways could be enhanced. Additionally, P14 observed that “the sentiment seems to affect the output by a large margin,” indicating that the weighting of the sentiment module may at times overly influence the system’s responses, an important design consideration for future refinement.

Participants also suggested specific interface and usability improvements. For example, P12 recommended adjusting button colors for better visibility, while P13 expressed a desire for a more readable output structure to improve comprehension of results. Despite these suggestions, users consistently recognized the system’s potential value. P11 highlighted that the empathic-CDSS’s “step-by-step recommendations” offer clear guidance, and P10 suggested that the system “may assist in improving care for a patient.” Regarding the XAI component, P14 stated: “The ability to understand how the model makes a decision is helpful for patients and doctors.” This feedback reflects users’ cautious optimism—while acknowledging areas for improvement, they recognize the importance and usefulness of transparent explanation in clinical decision support.

## Discussion

### Principal Findings

This study shows that the proposed empathic-CDSS, which integrates multimodal emotion perception with adaptive empathic response generation, outperformed the baseline CDSS that lacked emotion-awareness of the system’s user. The empathic-CDSS produced higher scores in usability, clarity, personalization, trust, and user satisfaction. These improvements are consistent with prior research indicating that emotionally adaptive technologies can reduce cognitive load, improve comprehension, and enhance trust in AI systems [40-44].

Our findings confirm that empathetic adaptation significantly enhances system credibility, reliability, and user satisfaction. When systems recognize user emotional states, users report higher levels of system confidence—aligning with the need for more human-centered clinical AI. The integration of real-time emotion perception significantly enhances system usability, clarity, and comprehensibility while boosting the personalization and relevance of insights generated by the system. By dynamically aligning explanation complexity with user emotional states, the system successfully translates technical XAI outputs into interpretable, plain-language clinical insights.

Higher scores in clarity and comprehensibility (mean 8.09, SD 1.53, vs 6.39, SD 2.00, *P*<.001), with a large effect size, demonstrate the substantial impact of this approach on user understanding. This represents a meaningful improvement in clinicians’ comprehension of model predictions rather than a marginal gain. These findings indicate that adaptive explanations with emotional perception not only enhance subjective satisfaction but also improve clinicians’ ability to interpret AI predictions. By integrating causal reasoning outputs with plain-language explanations generated by LLMs, the system facilitates a clearer understanding of predictive logic and enhances the interpretability of AI-assisted decision-making. LLMs serve as a bridge, translating complex SHAP feature weights and technical causal graphs into narrative lay summaries, making them more accessible to clinicians without deep AI expertise. This shift from static technical visualizations to dynamic empathetic dialogue effectively mitigates the “black box” nature of clinical AI, thereby enhancing trust and reliability. Prior work, consistent with our observations, indicates that emotion-adaptive explanation strategies can improve user engagement, reduce frustration, and lower perceived cognitive load during complex analytical interactions [45-48].

This study’s results are consistent with studies showing that empathic responses can stabilize emotional states and reduce interactional friction during demanding tasks [49]. Participants in our study frequently described the empathic-CDSS (vs basic system) as clearer, easier to engage with, and more understanding. These perceptions likely contributed to the significantly higher ratings of trustworthiness and reliability. Trust is widely recognized as a crucial factor for clinical adoption of AI systems [41,43].

### Comparison With Prior Work

Most existing CDSS and XAI frameworks focus on improving algorithmic transparency while paying little attention to how explanations are communicated to clinicians or system users [50-52]. In many systems, explanation generation is treated as a static process that delivers identical information regardless of user context or emotional state [51,53]. Our findings challenge this assumption. Consistent with recent work in affective computing and empathy-aware conversational agents [40-42], our study demonstrates that dynamically adapting the communication style of explanations can significantly improve user comprehension and interaction quality.

Unlike earlier empathic-CDSS prototypes that relied on rule-based or predefined text responses, our innovative system incorporates real-time arousal and valence detection. These signals allow the LLM to adjust explanation complexity, tone, and pacing in response to subtle emotional changes during interaction. This novel feature addresses a key limitation of static XAI approaches, which do not respond to users’ evolving cognitive or emotional states. Our findings also extend prior research by applying empathy-aware interaction to the problem of helping nontechnical users interpret machine-learning explanations, which represents a new and challenging scenario.

In addition, integrating XAI technology with emotion-aware LLM-driven communication enables more effective transmission of model reasoning processes to users. Real-time detection of emotional valence and arousal allows the system to dynamically adapt the level of detail, tone, and complexity of explanations to better match users’ cognitive and emotional states. This real-time adaptive explanation strategy enhances the clarity and comprehensibility of model outputs, as reflected in higher user ratings and qualitative feedback, and facilitates smoother and more effective clinical decision support interactions. Furthermore, the strong performance of the affect recognition module supports the reliability of emotion-adaptive explanation delivery.

As a result, a key contribution of this work is demonstrating that the delivery method of explanations is as important as the content itself. This contribution provides a concrete example of how multimodal emotion perception, XAI techniques, and LLM-based communication can work together to improve user understanding.

### Implications for LLM-Based Clinical Decision Support

This study also highlights a new role for LLMs in health care. LLMs can function as empathetic mediators between complex AI models and users. In addition to translating technical XAI outputs into natural language, the empathic-CDSS incorporates the user’s emotional state into the explanation process. This advance aligns with emerging evidence that emotionally intelligent conversational agents can enhance trust, adherence, and perceived clarity even when system users lack technical expertise [42-44].

Our results point toward a new model of clinician-AI collaboration. In this model, intelligent systems do not merely present information but actively sense cognitive or emotional load and adjust the way information is delivered. This creates opportunities for more effective and supportive interactions, particularly in high-stakes clinical environments.

By improving interpretability, reducing interaction difficulty, and providing emotionally aligned explanations, empathic XAI frameworks such as empathic-CDSS may help lower barriers to adoption of AI tools in behavioral health monitoring and intervention settings. By enhancing trust, usability, and perceived usefulness, these systems facilitate greater acceptance among clinicians who may otherwise be hesitant to adopt complex AI-driven decision support tools.

### Limitations and Future Work

Although this study achieved positive results, several limitations remain that need to be addressed in future work. First, the participant sample of system users consisted primarily of younger adults with high educational attainment, many of whom had limited or no real clinical experience. This demographic profile may not fully reflect practicing users who routinely engage with clinical decision support tools. Future studies should involve a more diverse user population, including physicians, nurses, and allied health professionals, in real-world clinical environments to evaluate performance, workload impact, and long-term adoption of CDSS. The age distribution in our sample was uneven, with no participants in the 40-49 years age group. Although no age-based exclusion criteria were applied, this gap likely reflects sampling variability given the modest sample size (N=33). As a result, findings may not fully generalize to midlife adults within this age range. Future studies with larger and more stratified samples are needed to examine potential age-related differences more comprehensively.

Second, the current system relies on commercial OpenAI application programming interfaces for emotion-adaptive response generation. Although effective for prototyping, this dependency raises concerns about response latency, the cost of large-scale deployment, and data privacy. Transmitting facial expressions and conversational content to external servers poses potential confidentiality risks even when anonymization is applied. To support future clinical translation, deployment using locally hosted or HIPAA (Health Insurance Portability and Accountability Act)-compliant open-source LLMs should be explored, along with on-device or edge-based emotion processing.

Third, while the emotion-perception module improved the clarity and smoothness of interactions overall, several users reported that empathic adjustments sometimes altered the specificity or focus of the explanations. For instance, some responses became overly supportive at the expense of addressing the clinical question directly. This suggests the need for finer control over the strength and timing of empathic behaviors. Future designs may include adjustable empathy levels, task-sensitive modes, or guardrails that ensure explanation fidelity is maintained.

Finally, emotional perception in the current system is based on facial arousal and valence estimation captured through the webcam. This approach may miss important cues such as vocal tone, hesitations, or physiological signals that often reflect cognitive load or frustration. Integrating multimodal data (for example, voice features or wearable sensor data) and developing reinforcement learning mechanisms that adapt emotion recognition based on user feedback could substantially improve model accuracy and the appropriateness of empathic responses.

Overall, these findings demonstrate both the potential benefits and limitations of integrating empathetic communication into AI-driven CDSSs. Key advantages include improved trust, enhanced understanding of model reasoning, and more effective user interaction. At the same time, these systems present important limitations, including potential variability in emotion recognition accuracy and the need to ensure that emotional adaptation does not compromise clinical precision or decision clarity in high-risk medical contexts. Future work should further refine these systems to better balance emotional responsiveness, interpretability, and clinical reliability.

### Conclusions

This study introduced an empathic-CDSS that integrates a continuous valence-arousal emotion model with LLMs to support affect-aware interaction in clinical decision support. By using real-time emotional cues to guide response generation, the system is able to detect subtle affective shifts and deliver explanations that are more contextually appropriate and emotionally aligned with the clinician’s affective state.

Our findings demonstrate the feasibility and value of combining emotion perception with LLM-based explanation generation. Participants rated the empathic-CDSS higher than the basic system across usability, clarity, personalization, trust, and satisfaction, highlighting the importance of emotion-aware communication in facilitating effective human-AI collaboration. This work suggests that the delivery and adaptation of explanations are as critical as their informational content, particularly when clinicians must interpret complex AI outputs under possible situations of high cognitive load and time pressure.

Overall, this research provides early evidence that emotionally intelligent conversational systems can enhance the clinician experience in AI-assisted decision-making. The integration of valence-arousal modeling with LLMs offers a promising direction for future emotionally aware clinical AI tools, and it lays the groundwork for designing more supportive, intuitive, and human-centered conversational interfaces in health care.

## Appendix

Multimedia Appendix 1 Methodological details.

Multimedia Appendix 2 Full facial behavior analysis feature set.

Multimedia Appendix 3 Survey questions.

## Abbreviations

AFEW-VA: Acted Facial Expressions in the Wild – Valence-Arousal (a publicly available dataset for affective computing)
AI: artificial intelligence
CDSS: clinical decision support system
Empathic-CDSS: empathic clinical decision support system
HIPAA: Health Insurance Portability and Accountability Act
LLM: large language model
MI: motivational interviewing
ML: machine learning
ReLU: rectified linear unit
SHAP: Shapley Additive Explanations
XAI: explainable artificial intelligence
