# Supplementary material for: Emotion-Adaptive Large Language Model–Driven Clinical Decision Support: User Evaluation of the Empathic Clinical Decision Support System Framework for Trust and Explainability
Source: JMIR Hum Factors. 2026 May 22;13:e89005. doi: 10.2196/89005 (PMC13241800; doi:10.2196/89005)
Supplement: Multimedia Appendix 1 [file humanfactors_v13i1e89005_app1.docx]

## Personalized Model Generation for Cannabis Users (“Patients”)

In this study, we employed a personalized model generation strategy to accurately predict outcomes for each individual cannabis user (“patients” in this CDSS), whereby we constructed a machine learning model that is exclusive to each cannabis user to ensure that the model accurately captures and reflects individual differences. This approach centers on generating a separate model for each person, rather than using a common model for all users’ data.

Our methodology focuses on constructing personalized models for each cannabis user to retrospectively analyze their historical data and identify factors associated with prior cannabis intoxication events. To this end, we utilize each user's data, which contains the labels required for supervised learning. This dataset first undergoes comprehensive preprocessing to ensure quality and consistency, including removing duplicate values, filling missing values, and performing feature normalization. To eliminate redundancy and prevent model overfitting, we subsequently calculate feature correlations and remove highly correlated variables. The finalized dataset is then employed to train the user's bespoke model. This model generates post-hoc insights and counterfactual explanations regarding the user's past behavior. This enables clinicians and users to gain a deeper understanding of the evidence base underpinning individual behavioral patterns, grounded in previously collected data. For example, in counterfactual analysis, we use decision tree regression models to train each clinician individually. This individual data-based training ensures that the model fully captures the clinician’s personalized characteristics and behavioral patterns, rather than being diluted by group characteristics. With this personalized model generation strategy, we not only improve the accuracy of model predictions, but also enhance the user’s trust in the model’s analytical results.

Each cannabis user’s model is constructed based on his or her unique data, with full consideration of individual differences, so that the model can better reflect the behavioral patterns and characteristic changes of cannabis users.

## Interpretability Analysis

Interpretability analysis aims to provide clinicians with an understanding of the behavior of machine learning models. By integrating SHAP Value Calculation [29], Causal Analysis [16], Counterfactual Analysis [14], and Rule Extraction [15], the system is able to explain the model’s decision-making process from multiple perspectives, enhancing the transparency and interpretability of the model.

Each explainability technique is selected based on its compatibility with the model used. SHAP value analysis is applied to the XGBoost model, leveraging its tree structure to effectively quantify the contribution of each feature to the prediction results. Counterfactual analysis is conducted using the Decision Tree model, which facilitates the generation of interpretable and reasonable counterfactual samples. Rule extraction employs the SkopeRules toolkit, which is based on decision tree methods and can extract human-readable logical rules from the model. Causal analysis is implemented using statistical methods to uncover potential causal relationships within the data. In order to make XAI easy to understand for the clinician, all XAI results have been represented as images.

SHAP values (Shapley Additive Explanations) are used to quantify the contribution of each input feature of the model to the prediction results. In this way, the system can show the clinician how the model makes decisions based on different features. The SHAP value of each feature reflects its positive or negative impact on the predicted outcome, and the clinician can use this analysis to identify which features are most important to the final decision. This process helps clinicians understand the model’s “black box” decision making and makes the model’s reasoning process more transparent and intuitive.

Through causal inference techniques, the system analyzes the causal relationships between variables, helping the clinician to identify the factors that have a significant impact on the predicted outcome [29]. Causal analysis allows clinicians to explore the logic behind the model’s predictions and understand how different features interact with each other to influence the final outcome. For example, the system can show the direct impact of changes in variables under different assumptions, allowing the clinician to not only see the results of the model predictions, but also understand the causal chain that led to those results [21].

Counterfactual analysis is another feature that the system provides to help clinicians understand the behavior of the model under different conditions. In counterfactual analysis, the system generates “what if” scenarios and shows how these scenarios affect the model’s output. Counterfactual samples are generated by fine-tuning or altering specific features, and the clinician can visualize how these changes affect the predictions. This analytics approach helps clinicians explore how the model performs in different scenarios to further enhance their understanding of the model. For example, the system uses a decision tree regression model to generate counterfactual samples, calculate changes in features, and show the most important influences [14]. In the process, the system calculates and ranks the amount of change in each feature based on the model’s predictions of the original and counterfactual data, demonstrating the top 20 most important features. By analyzing these features in depth, clinicians can understand how the changes predicted by the model occur under specific input conditions [30].

In addition to providing quantitative feature importance analysis, the system also extracts the model’s decision rules through the SkopeRules method. This method helps clinicians understand how the model makes predictions under specific conditions by generating simple and transparent rules. By refining the model’s decision paths, SkopeRules show under what circumstances the model triggers certain predictions, and these rules enable clinicians to understand the internal logic of complex models in a more intuitive way [15]. This rule-based explanation is particularly suitable for scenarios that require transparency in business logic, enabling clinicians to obtain a clear explanation of the model’s behavior.

This system combines a variety of interpretability techniques to help clinicians understand and scrutinize the behavior and decisions of machine learning models from multiple perspectives. Whether it is global feature importance analysis based on SHAP values, variable interaction impact analysis based on causal inference, hypothetical scenarios generated through counterfactuals, or decision rules extracted through SkopeRules, the system is committed to presenting complex model behaviors to the clinician in a clear and easy-to-understand manner. This multi-level explanatory analysis not only improves the transparency of the model but also enhances clinicians’ understanding of the model results.

Fine Tuning Model and Dual Language Model Architecture

The fine-tuned model serves as the system’s decision engine, synthesizing the XAI analysis results and the emotional context into a unified output. It combines the insights provided by the first model with the sentiment data to generate personalized and context-aware responses. When sentiment analysis indicates a negative state, the fine-tuned model adjusts tone and language to provide more support, simplify technical details, and reduce clinician anxiety. In the case of positive sentiment, the model provides encouraging and motivational feedback while providing deeper insights into the XAI results. For neutral sentiment, the model maintains a balanced and professional tone that ensures clarity and ease of understanding. By separating the responsibilities of the two models, the system achieves both technical rigor and emotional adaptability.

The standard GPT-4 ensures an accurate and precise interpretation of the XAI output, while the fine-tuned GPT-4 only processes the sentiment analysis results to generate a preliminary sentiment assessment and generate a dialogue prompt. This prompt is then passed to the standard GPT-4, which combines it with the clinician’s query and the XAI analysis results to generate the final response. This step-by-step approach ensures that the response is technically accurate and emotionally intelligent, thereby enhancing clinicians’ engagement and comprehension.

## Prompt Engineering

In this study, we design and develop a dialog system that is capable of dynamically adapting its responses to the emotional state of the clinician, leveraging prompt engineering methods [34] to optimize feedback in the dialog. The system integrates multiple high-level components, including emotion recognition and natural language processing models, to achieve its core functionality. One model detects the clinician’s emotional state through a combination of text sentiment analysis and facial expression recognition. Then, another model generates personalized and context-aware responses based on the detected emotion.

Additionally, in the explanation of XAI results, we avoided using specific numerical values and overly technical expressions, and instead used more general, qualitative descriptions to simplify complex concepts and make them easier for non-technical clinicians to understand. Instead of directly informing the clinicians of the weight that a feature contributes to the model output, the system uses language such as “this feature plays a more important role in the model’s decision” to express the importance of the feature. Similarly, when explaining the probability of a model’s output, the system uses terms such as “high likelihood” or “medium impact” instead of precise numerical values, thus making the explanation more approachable and easier to comprehend [35].

The core mechanism of the system involves perceiving the clinician’s emotional state and generating a tailored response that resonates with the clinician’s emotion. For clinicians experiencing negative emotions, the system adopts a compassionate and supportive tone, avoiding the use of complex technical terms. Conversely, when the clinician is in a positive emotional state, the system adopts an encouraging and motivating approach, acknowledging the clinician’s progress and providing deeper insights to promote further engagement. For neutral emotional states, the system maintains a friendly but professional tone, ensuring clarity and accessibility of explanations and encouraging the clinician to freely explore further queries.

The emotional prompt structure is designed to match the clinician’s emotion (defined by arousal and valence) with a tailored response generated by the system. Arousal (representing energy or intensity) and valence (indicating positivity or negativity) are used as the primary dimensions for categorizing the clinician’s emotional state. Each combination of arousal and valence is associated with a specific text polarity and corresponding strategy to provide empathetic and context-sensitive responses. For instance, if the system identifies low arousal and negative valence, with text sentiment marked as positive and the detected emotion being "angry," the prompt sent to the fine-tuned model would state: "The clinician appears low arousal, with a negative valence, and their text is positive. Detected emotion: Angry. Respond accordingly." This structured format ensures that the fine-tuned model has all the necessary contextual information to generate an appropriate, emotionally intelligent response without requiring the system to predetermine specific language or tone. By streamlining the emotional analysis into succinct prompts, the system leverages the fine-tuned model’s capabilities to dynamically adapt responses, ensuring they are empathetic, contextually relevant, and user-focused.

Technical Implementation

This research developed a web-based interactive application designed to provide explainable machine learning analytics, causal reasoning, and sentiment analysis services. The application allows clinicians to interact with the system via a natural language interface to access information such as model prediction explanations, causal reasoning insights, and counterfactual reasoning. These features are integrated into a user-friendly chat interface to enable non-technical clinicians to understand complex machine learning model decisions.

The system processes clinicians’ input through multiple integrated components. Text input is analyzed using the GPT-4 model provided by OpenAI [25], which excels at sentiment classification by identifying the emotional tone (positive, negative, or neutral) of a clinician’s message. At the same time, the system performs advanced sentiment analysis using emotional valence and arousal derived from camera-based facial expression recognition. This dual approach ensures an in-depth understanding of the clinician’s emotions. The results of these analyses, along with the answers generated by GPT-4, are further processed by a fine-tuned model to synthesize the output. This model integrates text sentiment, facial emotional valence, and arousal data to provide coherent and emotionally intelligent responses. To ensure transparency and with the clinician's full knowledge and consent, all clinician interactions are recorded for subsequent analysis and tracking, enabling continuous system improvement.

The main contribution of this research lies in the organic integration of the CDSS on the clinician-facing side regarding multimodal affective recognition, interpretable visualization of model results, and affective empathetic dialogue generation modules, achieving synergy from the dimensions of clinician experience and technical transparency. On the one hand, concerns, anxiety, or possible frustration that clinicians may have when facing complex AI models can be promptly identified and targeted to alleviate these negative experiences. On the other hand, XAI-based visualization and dialogue generation mechanisms ensure the transparency, controllability, and professionalism required of the system. Through this “joint system” design approach (i.e., affect recognition and XAI of model results), we have not only achieved emotionally sensitive interpretable AI, but also provided a replicable and extensible reference paradigm for intelligent systems for non-technical clinicians. In the future, we will further deepen our research in areas such as trusted human-machine collaboration, personalized customization of CDSS, and enhancing the system's ability to adapt effectively to a wide range of scenarios and contexts. We will also promote the application and implementation of emotional empathy and interpretability in more intelligent scenarios.
