# Supplementary material for: Emotion-Adaptive Large Language Model–Driven Clinical Decision Support: User Evaluation of the Empathic Clinical Decision Support System Framework for Trust and Explainability
Source: JMIR Hum Factors. 2026 May 22;13:e89005. doi: 10.2196/89005 (PMC13241800; doi:10.2196/89005)
Supplement: Multimedia Appendix 2 [file humanfactors_v13i1e89005_app2.docx]

| **Feature name** | **Value range / unit** | **Example value** | **Description** |
| --- | --- | --- | --- |
| Action Units (AU) | 0–1 | — | Intensity of specific facial muscle movements according to the Facial Action Coding System (FACS). |
| AU01 | 0–1 | 0.049 | Inner brow raiser |
| AU02 | 0–1 | 0.005 | Outer brow raiser |
| AU04 | 0–1 | 0.308 | Brow lowerer |
| AU06 | 0–1 | 0.305 | Cheek raiser |
| AU07 | 0–1 | 0.917 | Lid tightener |
| AU10 | 0–1 | 0.293 | Upper-lip raiser |
| AU12 | 0–1 | 0.886 | Lip corner puller |
| AU14 | 0–1 | 0.009 | Dimpler |
| AU15 | 0–1 | 0.497 | Lip corner depressor |
| AU17 | 0–1 | 0.304 | Chin raiser |
| AU23 | 0–1 | 0.046 | Lip tightener |
| AU24 | 0–1 | 0.004 | Lip pressor |
| Head pose | −180° – 180° | yaw = 12.4, pitch = 13.3, roll = 177.4 | Orientation of the head relative to the camera (horizontal, vertical, and tilt rotation). |
| Emotion probabilities | 0–1 | Angry = 0.43; Sad = 0.16; Neutral = 0.27 | Model-predicted likelihood for each of seven basic expressions (angry, disgust, fear, happy, sad, surprise, neutral). |
| Peripheral features | 0–1 | LeftEar = 0.19; RightEar = 0.24 | Visibility or geometric status of ear landmarks used for pose estimation. |
| Facial landmarks | Pixel coordinates (x,y) | (330, 168) – (521, 171) | 68 two-dimensional reference points mapping the facial contour, eyes, nose, and mouth for geometry and symmetry analysis. |
| landmark_0 – landmark_16 | — | — | Jawline contour points |
| landmark_17 – landmark_26 | — | — | Eyebrow contour points |
| landmark_27 – landmark_35 | — | — | Nose ridge and base |
| landmark_36 – landmark_47 | — | — | Eye region |
| landmark_48 – landmark_67 | — | — | Mouth and lip region |
