# Supplementary material for: Emotion-Adaptive Large Language Model–Driven Clinical Decision Support: User Evaluation of the Empathic Clinical Decision Support System Framework for Trust and Explainability
Source: JMIR Hum Factors. 2026 May 22;13:e89005. doi: 10.2196/89005 (PMC13241800; doi:10.2196/89005)
Supplement: Multimedia Appendix 3 [file humanfactors_v13i1e89005_app3.docx]

| **Section** | **Question** |
| --- | --- |
| Emotion and Stress | Q1: What is your current mood? Please select one of the following: Neutral, Happiness, Sadness, Anger, Fear, Disgust, or Surprise. [58]  Q2: What is your current stress level on a scale of 1 to 10? (1 being the lowest and 10 being the highest stress level.) [59] |
| System Usability [6] | Q1: How easy was it to interact with the system using natural language? (1 - Very Difficult, 10 - Very Easy)  Q2: How intuitive was the user interface for navigating through patient data and the system's insights? (1 - Not Intuitive, 10 - Very Intuitive)  Q3: How satisfied were you with the system’s response time in delivering insights? (1 - Very Dissatisfied, 10 - Very Satisfied)  Q4: Does the system's answer correctly interpret cannabis user data? (1 - Very Dissatisfied, 10 - Very Satisfied) |
| Personalization and Relevance [37] | Q1: How do system insights and recommendations correlate with improved personal health and wellness for patients? (1 - Not Relevant, 10 - Very Relevant)  Q2: To what extent did you feel the insights were personalized to cannabis patient's individual data and behaviors? (1 - Not Personalized, 10 - Highly Personalized)  Q3: Did the system adequately consider patient's current condition when providing insights (e.g., heart rate, sleep patterns)? (1 - Not at All, 10 - Completely) |
| Clarity and Comprehensibility [5] | Q1: How clear and understandable were the explanations provided by the system (e.g., SHAP values, causal diagrams)? (1 - Very Confusing, 10 - Very Clear)  Q2: How helpful were the system’s visualizations (e.g., SHAP plots, causal diagrams) in aiding your understanding of the results? (1 - Not Helpful, 10 - Very Helpful)  Q3: Did you find the response generated by the system easy to follow? (1 - Very Difficult, 10 - Very Easy)  Q4: Does this response contain evidence of correct personalization, reference appropriate user data, or correctly refuse to answer when such data is missing? (1 - Very Difficult, 10 - Very Easy)  Q5: Does the system's response demonstrate correct personalization, reference relevant user data appropriately, and correctly refuse to answer when the required data is missing? (1 - Very Difficult, 10 - Very Easy) |
| System Benefits [2] | Q1: How beneficial do you believe the insights were in improving your understanding of patient health behaviors (e.g., sleep, exercise)? (1 - Not Beneficial, 10 - Very Beneficial)  Q2: To what extent do you believe the system's recommendations will help you improve patient health outcomes (e.g., better sleep, increased physical activity)? (1 - Not Likely to Help, 10 - Very Likely to Help)  Q3: Would you recommend this system to others looking for patient health insights from wearable data? (1 - Definitely Not, 10 - Definitely Yes)  Q4: Did the system's response avoid misleading you? (1 - Definitely Not, 10 - Definitely Yes) |
| User Satisfaction [9,21] | Q1: How satisfied are you with the overall quality of the insights and recommendations provided by the system? (1 - Very Dissatisfied, 10 - Very Satisfied)  Q2: How likely are you to continue using this system to monitor and improve patient health in the future? (1 - Not Likely, 10 - Very Likely) |
| Trust and Reliability [22, 38] | Q1: Please rate the Perceived Trustworthiness of this system in meeting your needs and expectations: (1 being 'not trustworthy at all' and 10 being 'very trustworthy').  Q2: How would you rate the overall reliability of the system? (1 – not reliable, 10 – very reliable) |
| Open-Ended Questions | Q1: What specific aspect of the system did you find most valuable? [39]  Q2: Were there any areas where the system's insights or recommendations fell short of your expectations? [40]  Q3: Do you have any suggestions for improving the clarity, personalization, or usability of the system? [41]  Q4: Which aspects of the insights do you find most valuable for the patient? [42]  Q5: Do you feel that the recommendations accurately reflect the patient 's current condition based on the provided data? Why or why not? [43]  Q6: Please share any additional feedback or comments regarding your experience with the system. [39] |
